# Histological remodelling of demineralised bone matrix allograft in posterolateral fusion of the spine – an ex vivo study

**DOI:** 10.1186/1471-2482-13-58

**Published:** 2013-12-13

**Authors:** Samy Bouaicha, Brigitte von Rechenberg, Georg Osterhoff, Guido A Wanner, Hans-Peter Simmen, Clément ML Werner

**Affiliations:** 1 Division of Traumatology, University Hospital of Zurich, University of Zurich, Raemistrasse 100, Zurich 8091, Switzerland; 2 Musculoskeletal Research Unit, Equine Hospital, Vetsuisse Faculty ZH, University of Zurich, Zurich, Switzerland

**Keywords:** Spine fusion, Demineralised bone matrix, Histology, Allograft, Ex vivo

## Abstract

**Background:**

Demineralised bone matrix (DBM) has shown to be effective in enhancing posterior fusion of the spine. Several animal studies and clinical investigations in humans showed its successful remodelling. The use of allogenic matrix may decrease the need of autologous bone graft and therefore helps prevent corresponding donor site morbidity. Since DBM products are very expensive, the question arises, whether it is completely remodelled into new bone, and therefore truly is comparable to autologous cancellous bone graft. To our knowledge there is no report of a consecutive series of patients where ex vivo histological analysis after postero-lateral fusion of the spine was performed.

**Methods:**

Osseous biopsies of nine consecutive patients who underwent postero-lateral fusion of the spine for trauma were obtained at the time of elective removal of the hardware. Histological samples were then analyzed on ground and thin sections stained with toluidine blue and von Kossa stainings.

**Results:**

Time span between index operation and removal of the metal ranged between 6 and 18 month. Histological analysis showed good incorporation and overall remodelling of DBM into new bone in all patients. No foreign body reaction was visible and new bone formation progressed time dependently with DBM in situ. Four out of nine patients showed more than 50% new bone formation after one year.

**Conclusion:**

DBM shows good overall remodelling properties in histological analysis and therefore seems to be an effective adjunct in postero-lateral fusion of the spine. Furthermore, DBM substitution increases over time.

## Background

The current gold standard in thoraco-lumbar fusion surgery of the spine is posterolateral arthrodesis using autologous bone graft from the iliac crest in addition to instrumentation [1,2]. Although autologous bone grafts provide ideal biological properties, graft harvesting may cause severe donor site morbidity [3-8]. Therefore, spine surgeons increasingly tend to use allogenic bone graft as osteoconductive and osteoinductive carriers enhancing solid fusion without disadvantages of graft harvesting. Demineralised bone matrix (DBM) has shown to be a reliable alternative in terms of its fusion capacities in several animal models as well as in clinical investigations in humans [9-17]. While histological performance of DBM was extensively investigated in animal fusion models [9-14], only few ex vivo studies were reported proving efficacy of this generally very expensive material. These studies dealt either with dental surgery procedures or idiopathic scoliosis in a child, whereas no ex vivo investigation was reported focusing on adult spine surgery [18,19]. To our knowledge, the present study is the first report of histological performance of DBM in a consecutive series of patients who underwent posterolateral fusion of the spine. The goal of our investigation therefore was to clarify whether DBM incorporates to the surrounding bone or not. The hypothesis was set up that transformation of DBM is related to its time in situ (“dwell time”).

## Methods

### Patients

In a retrospective study design a series of patients who underwent dorsal instrumentation and postero-lateral fusion of the thoraco-lumbar spine using DBM as bone substitute and subsequently were scheduled for elective removal of dorsal instrumentation hardware were included in our study. Patients with diagnosed systemic infection and septic loosening were excluded. Patients routinely were asked before surgery if a sample of the bony debris removed during uncovering of the implants may be used for histological analysis instead of been thrown away. Formal ethical approval of waste by-product analysis was not necessary. Still, all patients gave their written informed consent. At the time of hardware removal, small osseous biopsies then were obtained as by-product while uncovering the metallic rods.

### Histology

All bone samples were processed equally with primary fixation in 12% formalin solution. After ascending alcohol series and degreasing by xylene under vacuum conditions, the non-decalcified specimens were infiltrated and embedded in polymethylmetacrylate as described previously [20,21]. Ground (ca. 40 μm) and thin (5 μm) sections were prepared and (surface-) stained with either toluidin blue (TB) or von Kossa/McNeal (only thin sections). Histological sections were evaluated qualitatively and semiquantitatively. The latter was performed in the van-Kossa/McNeill sections, where DBM could well be distinguished from the newly formed bone and adjacent soft tissue. Semi-quantitative scores were given indicating the percentage of remaining DBM, resp. new bone formation (Table 1).

**Table 1:** Semi-quantitative histological evaluation

**Patient**	**Age**	**Fusion levels**	**DBX® in situ (month)**	**DBX® residuals (+/++/+++/++++)**	**New bone formation (+/++/+++/+++++)**
1	31	T10-T12	7	+	+++
2	52	T11-L1	18	-	++++
3	50	T11-L1	8	+	+++
4	39	T12-L2	9	++	++
5	39	T12-L2	8	++	++
6	64	T5-T9	8	+++	+
7	30	T12-L2	17	-	++++
8	46	T2-T5	6	++	++
9	48	T11-L1	9	++	++

Table 1: Case related histological analysis with scores: (+) = <25%, (++) = <26-50%, (+++) = <51-75% and (++++) < 76-100% indicating relationship between DBM and newly formed bone.

## Results

Nine patients (6 male and 3 female, 30 to 64 years old) that were scheduled for elective removal of dorsal instrumentation hardware over a 12-month time period were included in our study. Indications for hardware removal was subjectively disturbing hardware and the explicit patients wish to remove the implants in 8 out of nine patients. In one patient aseptic implant loosening was diagnosed. Osseous fusion was confirmed radiologically in all cases. For index operation 10 cc of DBX Mix® (SYNTHES, Oberdorf, Switzerland), a demineralised bone matrix substitute embedded in sodium hyaluronate mixed with cortical allograft bone chips, was used. The fusion bed was prepared using a high speed drill before DBX Mix® was added. No additional autologous bone graft harvesting of the iliac crest was conducted in all cases. Except of one female patient who presented pathological osteoporotic fracture with consecutive stenosis of the spinal canal, all other patients were initially operated for unstable traumatic vertebral fractures and healthy otherwise.

The time period between index operation and hardware removal and osseous biopsies respectively, was between 6 and 18 month (mean 10 month). No intraoperative and no postoperative complication including infection or extensive hematoma formation occurred in any patient. At the time of hardware removal, intraoperative testing showed solid fusion of all bridged segments in all cases and DBX Mix® seemed to be remodelled and integrated within the fusion mass.

**Figure 1:**
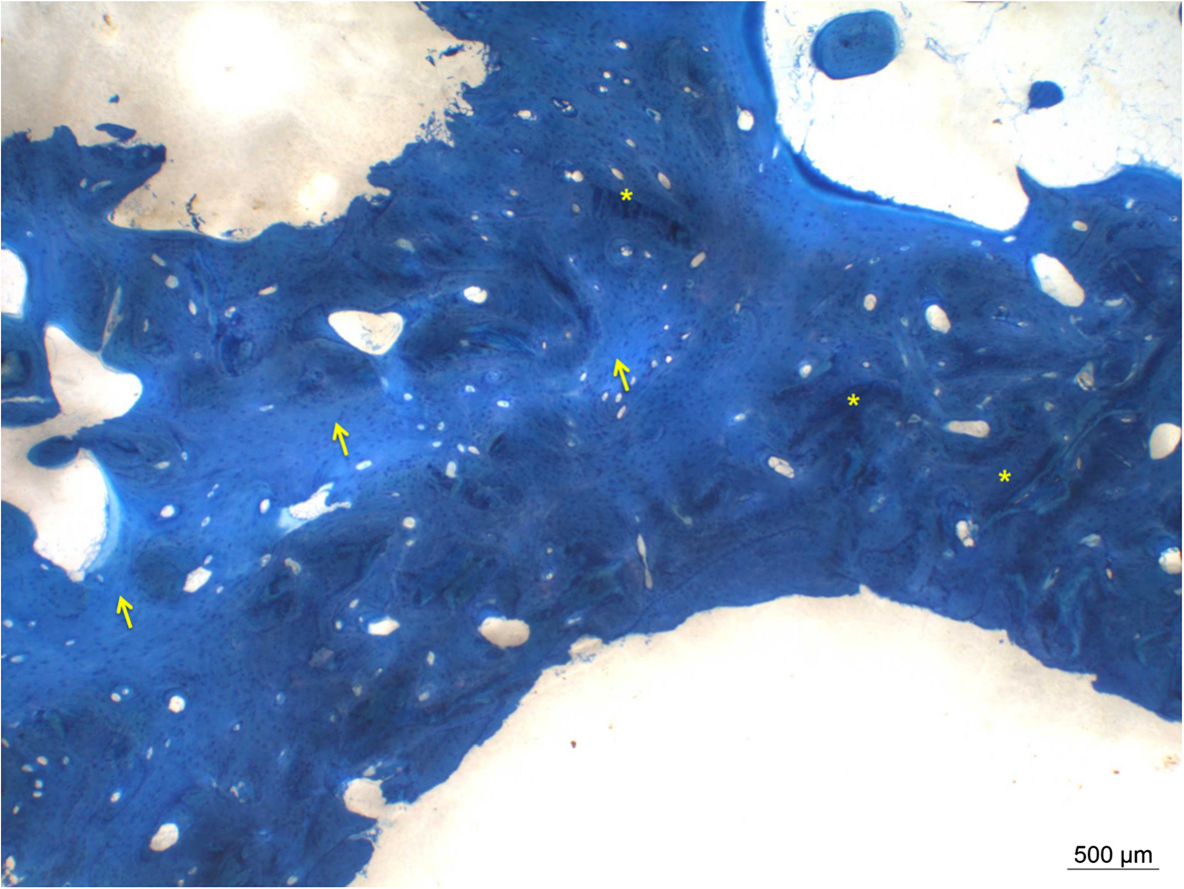
**Complete transformation of DBX® into new bone in a patient with in situ for 18 month.** Dark blue bone represents new woven bone matrix (*), lighter blue is already remodeled lamellar bone (arrow) (ground sections, PMMA, staining toluidine blue surface staining).

**Figure 2:**
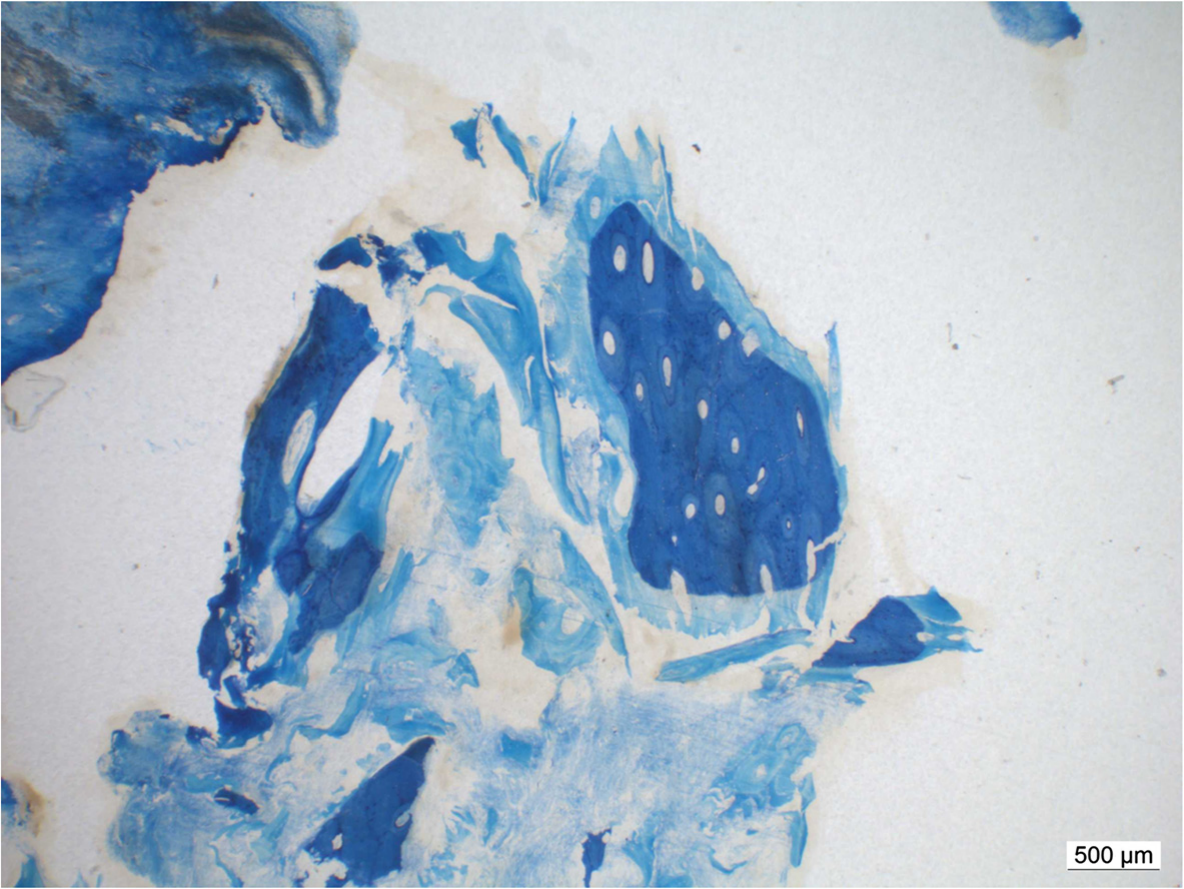
**Partial transformation of DBX® into new bone in a patient with in situ for 8 month.** The DBX® residuals (light blue) are still visible.

**Figure 3:**
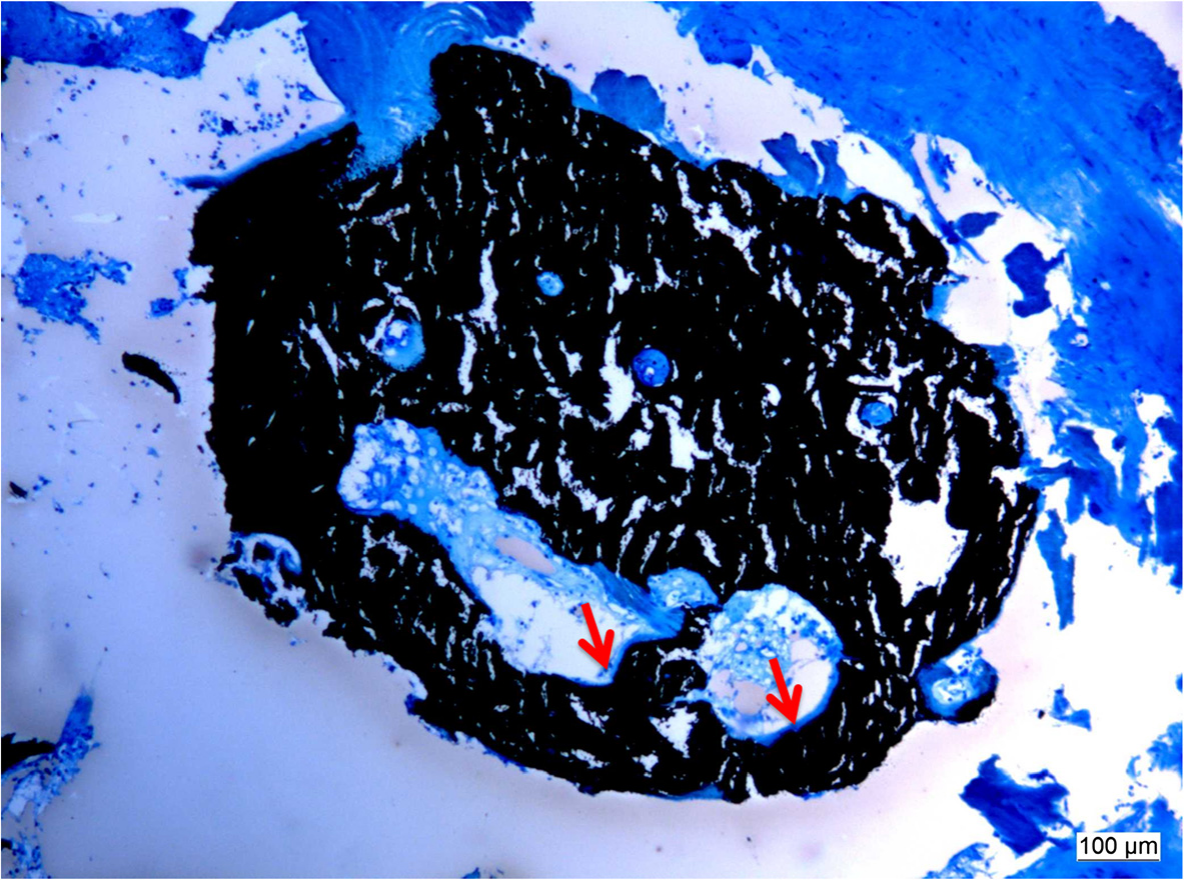
**«Creeping substitution»: demineralized bone matrix is slowly resorbed and simultaneously replaced by new bone.** Blacks indicates already mineralized bone matrix, turquoise pictures osteoid seams (arrows) (5 μm sections, PMMA, Staining von Kossa/McNeall).

**Figure 4:**
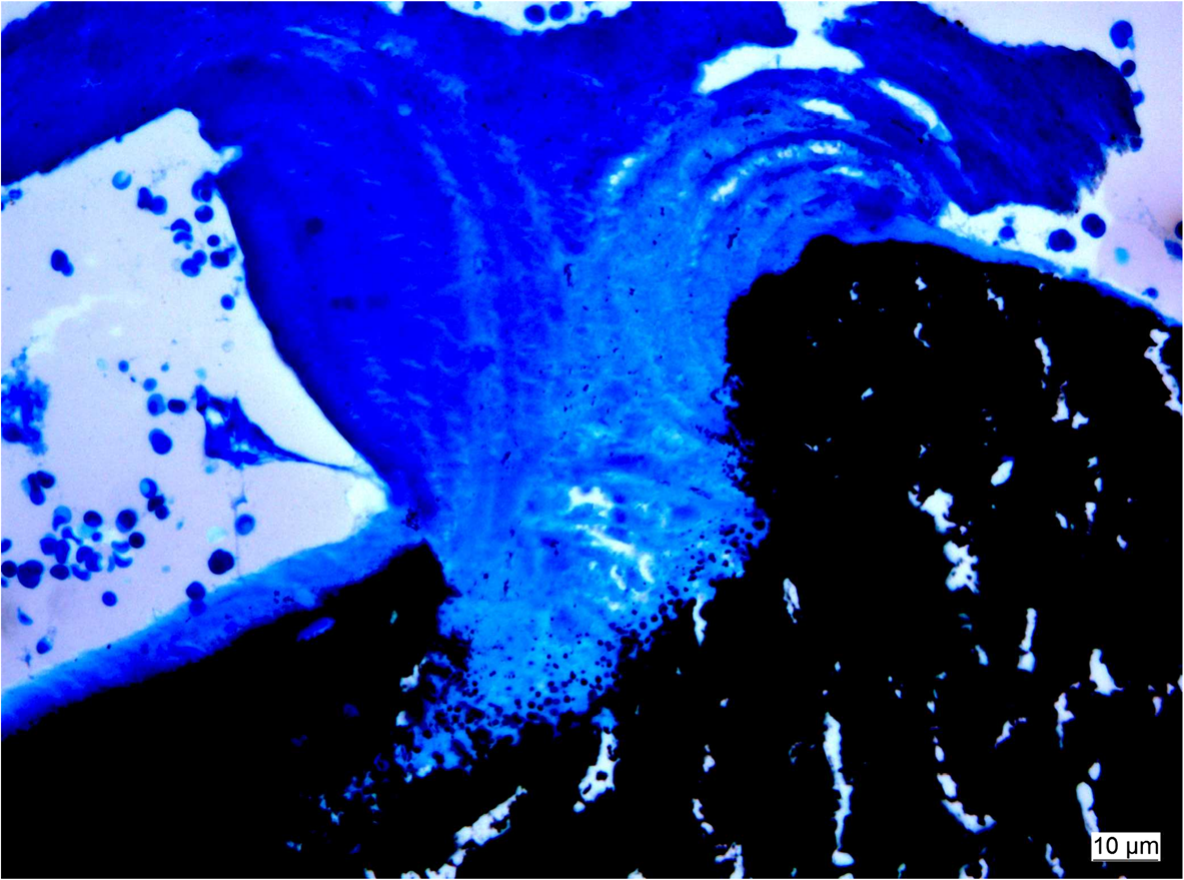
**Extensive matrix calcification (black) during the substitution process.** Note precipitation of calcium at the border between remaining matrix and newly formed bone (5 μm section, PMMA, Staining von Kossa/McNeall).

Qualitative and semi-quantitative, histological analysis showed time dependent partial remodelling in some samples and almost complete remodelling of the DBM into new bone in other cases. Detailed assessment showed not only reorganization of demineralised matrix but also of the autologous and allogenic bone chips generated through preparing the graft bed during initial surgery. While samples of patients with shorter “dwell time” of DBM in situ showed less new bone formation and more DBM and chips residuals, samples with longer stays showed almost complete substitution of DBM with a lot of new bone mineralization and progressive remodelling of cortical chips (see Figures 1 and 2 and Table 1). Figure 3 shows the “creeping substitution” [22] in a case of 6 month DBM in situ. Precipitation of new osteoid and extensive matrix calcification is shown in Figure 4. On the thin slices different phases of the reorganizational process could prove osteoinductive and osteoconductive capacities of the DBM. For case related analysis see also Table 1.

## Discussion

Since DBM for clinical use is very expensive compared to autologous bone grafting, effectiveness of such products is mandatory. While DBM gel as an autograft extender showed improved fusion in dogs [7], heterologous DBM provided equal fusion capacity as autologous bone graft in rats [8]. Comparable results were also obtained when DBM was used as partial substitute of autologous bone graft in a postero-lateral fusion model in rabbits [9]. In another rabbit model, DBM showed osteoinductive ability, while allogenic deep-frozen cortical bone did not [10]. Radiographic investigations in humans showed equal fusion rates of postero-lateral arthrodesis performed in patients with local autologous bone graft augmented with DBM and in those with autologous bone graft from the iliac crest alone [13]. Radiographic progression of the fusion with time was also shown when DBM was used as bone graft extender to decrease the need of autologous bone graft [14]. Intra-individual comparison of DBM augmented autograft at one side and autograft without adjunct at the contralateral side in patients who underwent postero-lateral fusion showed equal results in radiographic assessment [15]. An ex vivo histological analysis of different types of DBM products used for sinus lift procedures in oral surgery showed superiority of DBX® in terms of new bone formation and low residual demineralised matrix compared to other products [16]. In a recently published case report of a 7-year old male child suffering from idiopathic scoliosis who underwent posterior spinal fusion, DBM was used as sole graft source. Eleven month later on the basis of a routine surgical exploration, osseous biopsies were taken from the fusion site and histological analysis was performed. In this case, investigators found only mature bone with no residual DBM graft and concluded therefore DBM to be fully incorporated in the fusion mass [17].

Our results tend to support these overall findings of reliable remodelling of DBM to new bone. The histological analysis showed significant new bone formation and decreasing residual DBM material in all of our patients depending on the time span DBM was in situ. Additionally, cortical bone chips converted as well in the same manner. The verifiable process of “creeping substitution” allowed confirming active incorporation and reorganisation of the demineralised matrix.

Further analysis should be made to determine whether additional cortical bone chips are necessary to enhance new bone formation or if DBM putty alone provides best performance in postero-lateral fusion of the spine. Comparative study designs will help to analyse the bone remodelling patterns of different treatment courses such as the use of DBM versus autologous bone grafting or spontaneous fusion based on posttraumatic hematoma.

Limitations of our study are the small number of patients included in the collective and the lack of quantitative morphometric analysis based on standardized bone samples, which of course was not applicable in the clinical setting. Furthermore the results of our investigation are not applicable to postero-lateral fusion in general since our study population is relatively youg (mean age 44 years) and the reason for a fusion was trauma in eight of nine cases. Additionally there may be a bias with regard to the harvesting site of the bony samples, which were all taken around the rods during implant removal and therefore may not be representative for all fusion areas.

## Conclusion

DBX Mix® shows reliable remodelling in histological analysis and therefore seems to be an effective in postero-lateral fusion of the spine in the presented study population. Furthermore, DBM substitution increases over time.

## Competing interests

The authors declare that they have no competing interests.

## Authors’ contributions

SB performed data collection and analysis including interpretation of histological cuts and drafting of the manuscript. BvR processed the DBX tissue, introduced the semi-quantitative scoring of DBX histology and contributed significantly to the manuscript drafting. GO was involved in the interpretation of the data, drafting of the manuscript, and revised it critically for the intellectual content. GAW and H-PS have read, edited and approved the final manuscript. CMLW set up the study design, was the operating surgeon in all cases and revised the manuscript critically for the intellectual content to its final version. All authors read and approved the final manuscript.

## Pre-publication history

The pre-publication history for this paper can be accessed here:

http://www.biomedcentral.com/1471-2482/13/58/prepub

## Study design

Retrospective case series.
